# Advancing Biosensing Through Artificial Intelligence: Overcoming Conventional Limitations Towards Intelligent Diagnostic Systems

**DOI:** 10.3390/bios16100537

**Published:** 2026-09-25

**Authors:** Tarika Devi Vijayaraj, Rakshitha Karpagarajan, Sairam B. Subramaniam, Durgashini Annamalai, Swati Kumari

**Affiliations:** 1 Department of Microbiology, Sri Ramachandra Medical College & Research Institute (SRMC&RI), Sri Ramachandra Institute of Higher Education and Research, Chennai 600116, Indiam0724001@sriher.edu.in (R.K.);; 2 Department of Computer Science and Medical Engineering, Sri Ramachandra Faculty of Engineering and Technology (SRET), Sri Ramachandra Institute of Higher Education and Research, Chennai 600116, India

**Keywords:** biosensors, artificial intelligence, machine learning, intelligent biosensing, limit of detection, clinical actionability, clinical validation

## Abstract

Artificial intelligence (AI) is increasingly integrated with biosensors to improve signal processing, feature extraction, calibration, and clinical interpretation. However, analytical sensitivity and high internal validation accuracy do not necessarily translate into clinically useful or generalisable diagnostic performance. This review therefore reframes AI-enabled biosensing around clinical actionability rather than technological performance alone. We critically examine how AI can address signal noise, drift, matrix effects, and complex or high-dimensional biosensor outputs while evaluating how model selection should reflect data modality, dataset size, computational constraints, and intended deployment. Importantly, we distinguish the physical limit of detection (LoD) of the sensing system from algorithm-assisted effective detectability: AI cannot intrinsically lower the physical detection limit of a fixed transducer, but it can improve extraction and interpretation of weak signals near the noise floor. A central contribution of this review is a clinically actionable threshold framework that links representative biomarkers to decision-relevant concentration ranges, clarifying when further LoD reduction is clinically meaningful and when robustness, selectivity, dynamic range, calibration stability, and reproducibility matter more. We also critically appraise representative AI-enabled biosensor studies by sample size, validation strategy, reference standard, generalisability, interpretability, computational requirements, and evidence maturity, rather than headline accuracy alone. Across electrochemical, optical and spectroscopic, wearable, and point-of-care platforms, major translational barriers include biofouling, matrix interference, signal drift, dataset shift, insufficient external validation, privacy, and regulatory requirements. We conclude that progress in intelligent biosensing should be judged by reliable performance across clinically relevant ranges and demonstrated utility in real-world diagnostic workflows, rather than by progressively lower LoDs or isolated accuracy values alone.

## 1. Background

Biosensors are analytical systems that integrate biological recognition with physicochemical transduction to convert a biological interaction into a measurable signal. Modern electrochemical biosensing is often traced to Leland C. Clark Jr.’s membrane-covered oxygen electrode in the 1950s, which provided the electrochemical basis for the subsequent enzyme–electrode concept. In 1962, Clark and Lyons described an enzyme electrode for continuous glucose monitoring by coupling glucose oxidase-mediated glucose recognition with electrochemical oxygen measurement [1,2]. This work laid the foundation for modern electrochemical biosensing. Subsequent generations of enzyme biosensors evolved from systems that rely on naturally occurring electron acceptors, such as oxygen, to second-generation devices that use artificial redox mediators and third-generation approaches based on direct electron transfer between the redox-active biomolecule and the electrode [3].

Advances in nanomaterials, microfabrication, flexible electronics, wireless communication, and digital processing have substantially broadened the capabilities and applications of biosensors [4,5,6,7]. Biosensing platforms are now used for biomedical diagnosis and monitoring, point-of-care testing, drug discovery, food safety assessment, environmental monitoring, and detection of biological or chemical analytes [1,4]. Wearable systems have further extended biosensing from episodic measurements to longitudinal monitoring of physiological and biochemical parameters. Current platforms include continuous glucose monitors and devices that measure electrocardiographic, cardiovascular, mechanical, and biochemical signals, with increasing integration into smartphones and connected health systems [5,6,7].

Despite these technological advances, substantial clinical need remains. Noncommunicable diseases (NCDs), including cardiovascular diseases and diabetes, account for a major proportion of global mortality; the WHO estimates that NCDs caused at least 43 million deaths in 2021, including 18 million deaths before age 70 [8]. Cardiovascular diseases account for the largest share of NCD deaths, while diabetes and its complications also contribute substantially to morbidity and premature mortality [8,9]. Early recognition remains important because some disorders can remain clinically inconspicuous for prolonged periods; for example, symptoms of type 2 diabetes may be mild for years, and diagnosis may occur only after complications have developed [9]. At the same time, emerging and re-emerging infectious diseases continue to pose epidemic and pandemic risks, reinforcing the need for timely detection and surveillance [10].

Conventional laboratory diagnostics remain indispensable reference methods, but depending on the test and healthcare setting, they may require centralised infrastructure, trained personnel, sample transport, multiple processing steps, and turnaround times that limit immediate decision-making. Point-of-care biosensing can complement centralised testing by moving selected measurements closer to the patient, including in emergency departments, primary care facilities, community settings, and the home [11,12]. However, miniaturisation or analytical sensitivity alone does not ensure clinical utility. Translation of biosensors from controlled laboratory conditions to clinical samples is challenged by matrix interference, biofouling, signal variability, sensor drift, calibration instability, manufacturing variation, and heterogeneity among patients and specimens [12,13].

The integration of artificial intelligence (AI) and machine learning (ML) provides an additional computational layer to address some of these challenges. Depending on the sensing modality and clinical task, AI can support signal preprocessing, feature extraction, pattern recognition, classification, regression, calibration, anomaly detection, multimodal data integration, and decision support [7,11,12,14]. Importantly, AI does not compensate for an inadequately designed or unstable sensor; rather, its value depends on the quality and representativeness of the biosensor-derived input data and on appropriate validation of the complete sensor–algorithm system [11,12,13,14].

Beyond summarising biosensor platforms and AI methods, this review evaluates AI-enabled biosensing from a clinical and translational perspective. It places particular emphasis on distinguishing an ultra-low analytical limit of detection (LoD) from reliable performance across clinically relevant biomarker concentrations. The review further distinguishes the physicochemical LoD of the underlying sensing system from AI-assisted effective detectability, in which computational analysis may extract diagnostically useful information from weak, noisy, multidimensional, or otherwise difficult-to-interpret signals [14]. Rather than treating high accuracy or an increasingly low LoD as sufficient evidence of clinical readiness, representative studies are critically evaluated according to dataset size and independence, validation strategy, external generalisability, calibration, interpretability, computational requirements, reference standards, and clinical relevance. The central argument is that progress in AI-enabled biosensing should be assessed increasingly by clinical actionability, reproducibility, robustness, and external validation, rather than technological performance alone.

### Review Scope and Literature Selection Approach

This article was conducted as a structured narrative review with a targeted critical appraisal of representative original studies. The relevant literature was identified through searches of PubMed/MEDLINE, Scopus and Web of Science from database inception to 31 August 2026, supplemented by Google Scholar, backward reference list screening and forward citation tracking. The recent literature from 2022 to 2026 was prioritised for rapidly evolving areas of AI-enabled biosensing, validation, deployment and medical AI reporting, while older landmark publications were retained where historically or methodologically relevant. Primary studies included in the critical evidence synthesis were selected when an identifiable sensing platform or sensor-derived signal was analysed using an explicit AI/ML method and sufficient information was available regarding dataset or sample size, validation strategy and performance. Representative studies were purposively selected to span different sensing modalities and levels of evidence maturity rather than to constitute an exhaustive systematic sample. Detailed search concepts and study selection criteria are provided in Supplementary Table S1.

## 2. Biosensor Architecture and Data Characteristics Relevant to Artificial Intelligence

### 2.1. Core Biosensor Architecture

A biosensor is an analytical device that couples a biological recognition element with a physicochemical transduction mechanism to generate qualitative or quantitative information about a target analyte [1,15,16]. The target analyte interacts with a biorecognition element, which may include an enzyme, antibody, aptamer, receptor, nucleic acid, cell, tissue-derived component, or other biological recognition system. The transducer converts this biorecognition event into a measurable electrical, optical, mechanical, or thermal signal. Signal conditioning electronics may subsequently amplify or filter the response, after which analogue-to-digital conversion and computational processing enable the signal to be displayed, stored, transmitted, or further analysed [1,16]. The terminology and architecture are consistent with the International Union of Pure and Applied Chemistry (IUPAC) definition of biosensors and its recommendations for integrated biosensor systems [15,16]. The overall biosensor architecture and potential points of AI integration are illustrated in Figure 1.

In AI-enabled biosensing, computational analysis extends this conventional sensing workflow beyond signal acquisition and display. AI and machine learning (ML) approaches can be incorporated after digitisation or at multiple stages of the analytical pipeline to support noise reduction, drift correction, calibration, feature extraction, pattern recognition, regression, classification, anomaly detection, and clinical decision support [11,12,14]. Consequently, selection of an AI model should take into account the characteristics of the sensor output, including dimensionality, temporal structure, signal-to-noise ratio, drift, dataset size, label availability, and computational constraints, rather than algorithmic complexity alone [12,14].

### 2.2. Transduction Modalities and AI-Relevant Data Characteristics

Biosensors may be classified according to their transduction mechanism, which determines both the physical signal generated by the sensor and the structure of the data available for computational analysis [1,16,17]. Electrochemical biosensors generate measurements such as current, potential, or impedance; optical biosensors measure changes in properties such as fluorescence, absorbance, reflectance, or refractive index; piezoelectric and mechanical biosensors detect changes in frequency, resonance, mass loading, or mechanical response; and thermal biosensors measure heat or temperature changes associated with biochemical reactions [1,16,17]. Spectroscopic techniques such as Raman spectroscopy and surface-enhanced Raman spectroscopy (SERS) fall within the broader optical category but are distinguished here because they generate high-dimensional spectral data that present specific opportunities and challenges for AI analysis. Table 1 summarises the principal biosensor transduction modalities and their AI-relevant data characteristics.

The suitability of an AI architecture depends strongly on both the biosensor output and the intended analytical task. Conventional ML approaches may be preferable for relatively small, structured datasets in which informative features can be defined in advance, whereas deep learning architectures become increasingly useful when sufficiently large datasets contain complex images, spectra, waveforms, or multichannel time series signals [12,14]. High dimensionality alone does not justify deep learning; dataset size, overfitting risk, interpretability requirements, computational resources, latency, and the intended clinical deployment environment should also guide model selection [12,14]. Recent work on ML-assisted electrochemical biosensors similarly emphasises the importance of data processing for noisy, variable and continuous sensor outputs.

### 2.3. Deployment Formats and Computational Considerations

Biosensor deployment can be considered along two related but distinct axes: physical integration with the user, including wearable, implantable and ingestible devices, and clinical setting, including point-of-care, home-based and remote monitoring applications. These categories should not be treated as interchangeable because, for example, a wearable biosensor may operate at home or within a clinical monitoring programme, whereas a point-of-care biosensor may be handheld, benchtop, disposable or wearable.

Wearable biosensors enable continuous or repeated acquisition of physiological and biochemical measurements and increasingly support monitoring of biofluids such as sweat, tears, saliva, and interstitial fluid [5,6,7,18,19]. Their integration with smartphones and wireless healthcare systems permits longitudinal data collection and remote monitoring. From an AI perspective, however, continuous wearable signals introduce challenges related to motion artefacts, intermittent measurements, baseline drift, inter-individual variability, changing sensor–skin contact, environmental conditions, and limited power and memory. AI models intended for wearable deployment must therefore balance predictive performance with latency, memory requirements, energy consumption, connectivity, and robustness to noisy or incomplete real-world data [7,18,19]. Continuous wearable systems also place particular importance on long-term signal fidelity and embedded computational efficiency.

Implantable biosensors provide the opportunity for prolonged in vivo measurement and longitudinal monitoring but introduce additional requirements related to biocompatibility, foreign body responses, mechanical compatibility, signal stability, device longevity, and communication [20,21]. Long-term implantation can alter the sensor–tissue interface and contribute to signal drift or degradation, meaning that AI-based calibration or drift management strategies should be evaluated together with the physical stability of the sensing interface. Computational systems associated with implantable devices must also operate within constraints related to power availability, processing capacity, wireless communication, and long-term safety [20,21]. Importantly, computational correction cannot compensate for an irreversibly degraded or physiologically uninformative sensor signal.

Ingestible sensors provide temporary access to the gastrointestinal environment and have been investigated for monitoring gastrointestinal parameters, imaging, luminal chemistry, gastrointestinal bleeding, and medication adherence [22,23,24]. Kalantar-Zadeh et al. described ingestible sensing platforms capable of obtaining information from within the gastrointestinal tract, while Mimee et al. demonstrated an ingestible bacterial electronic system for gastrointestinal bleeding detection, and Chai et al. described an ingestible system for monitoring medication adherence [22,23,24]. Because ingestible devices generally have constrained operating periods, communication bandwidth, physical dimensions, and power availability, computational analysis may be distributed between the ingestible device, a nearby receiver or smartphone, and external computing infrastructure.

Point-of-care biosensors should be considered separately from body-worn deployment formats. Their defining requirement is that testing occurs at or near the site of clinical decision-making. AI integration may assist in interpreting complex electrochemical, optical, colorimetric, microfluidic, or wearable sensor outputs, but the clinical value of such systems also depends on turnaround time, ease of operation, robustness in real samples, computational requirements, and comparison with an appropriate reference method [11,12]. Thus, an algorithm that performs well on retrospective laboratory data may not necessarily be suitable for a resource-constrained point-of-care device.

Across these deployment formats, the optimal computational strategy is therefore determined by the interaction among data complexity, required inference latency, available memory and processing resources, energy consumption, connectivity, privacy requirements, sensor stability, and intended clinical purpose. Model selection should be performed in the context of the complete biosensor system rather than on predictive accuracy alone.

### 2.4. Advanced and Miniaturised Biosensing Platforms

Advances in microfluidics and miniaturisation have enabled lab-on-chip systems to integrate multiple analytical operations within compact platforms. Depending on the device, sample handling, reagent manipulation, separation, reaction, detection, and multiplexed analysis can be incorporated within a single microfluidic system, reducing sample and reagent requirements and supporting point-of-care operation [25]. Integrated microfluidic systems are particularly relevant to AI when they generate multidimensional electrochemical, optical, image-based, or multiplexed outputs that require simultaneous interpretation.

Nano-biosensors incorporate nanostructured materials such as metallic nanoparticles, carbon nanotubes, graphene-based materials, quantum dots, metal or metal oxide nanostructures, and magnetic nanoparticles to modify surface area, electron transfer behaviour, optical properties, molecular interactions, or signal transduction [4,26,27]. These approaches can improve specific analytical characteristics, but the benefits depend heavily on sensor design, recognition chemistry, sample matrix, fabrication reproducibility, and the intended application. Clinical translation therefore requires more than improved analytical sensitivity. Recent assessments of nano-biosensing emphasise the continuing need to improve reproducibility, usability, scalability, robustness and real-world performance.

From an AI perspective, advanced electrochemical, optical, spectroscopic, nanomaterial-based and microfluidic systems can generate multivariate signals that may be difficult to interpret using a single threshold or univariate calibration curve. AI can support preprocessing, multivariate calibration, feature extraction, pattern recognition, classification, regression and integration of multiple sensing channels [11,12,14,25]. However, increased signal complexity or improved analytical sensitivity does not itself establish clinical utility. Advanced platforms should also demonstrate reproducibility across devices and batches, robustness in relevant biological matrices, calibration stability, appropriate external validation, and reliable performance across the concentration range relevant to the intended clinical decision [14,25,27].

## 3. Artificial Intelligence in Biosensing

### 3.1. Role of Artificial Intelligence in Biosensing

Artificial intelligence encompasses computational approaches that identify patterns, generate predictions, or support decision-making from data. Machine learning (ML) refers to algorithms that learn relationships from data, whereas deep learning (DL) uses multilayer neural networks to learn hierarchical representations through successive nonlinear transformations [28]. In biosensing, AI can be incorporated at different stages of the analytical pipeline, including signal preprocessing, noise reduction, feature extraction, calibration, classification, anomaly detection, multimodal data integration, and clinical decision support.

The relevance of AI to biosensing is therefore not simply the use of increasingly complex algorithms. Its value depends on whether the selected model addresses a specific limitation of the sensing platform, such as signal noise, nonlinear sensor responses, drift, overlapping spectral features, high-dimensional data, or inter-individual variability. Model selection should therefore be guided by biosensor data characteristics, dataset size, intended clinical task, interpretability requirements, and computational constraints [14,28].

### 3.2. Selection of AI Approaches According to Biosensor Data

No single AI model is optimal for all biosensing applications. Different sensing modalities generate fundamentally different types of data, ranging from low-dimensional electrochemical measurements to continuous physiological time series, images, and high-dimensional spectroscopic profiles. The appropriate model should therefore be selected according to the structure of the biosensor output and the intended analytical task.

Traditional ML methods such as logistic regression, support vector machines, random forests, and gradient-boosting algorithms are particularly useful for relatively small or structured biosensor datasets. These approaches can support classification and regression while generally requiring fewer computational resources than deep neural networks. They may therefore be appropriate when sample sizes are limited or interpretability and deployment efficiency are important [14,28].

Deep learning approaches become more useful when biosensors generate complex or high-dimensional signals. CNNs can analyse one-dimensional time series signals or two-dimensional image and spectral representations by learning local features through convolutional filters. RNNs and LSTMs process sequential measurements by maintaining information across successive time points and are therefore applicable to continuous physiological monitoring. Transformers use self-attention to model relationships between different positions within a sequence and can capture long-range dependencies in complex time series, spectral, or multimodal datasets. Autoencoders learn compressed representations of input data and may be used for denoising, anomaly detection, dimensionality reduction, or representation learning [28].

Importantly, increasing model complexity also increases requirements for training data, memory, computation, and validation. Deep models may therefore be inappropriate for small biosensor datasets even when they achieve high internal accuracy. Conversely, wearable or point-of-care systems may require lightweight models capable of low-latency inference on devices with limited power and memory. The choice of AI architecture should therefore reflect both analytical performance and the intended deployment environment [14,28]. A practical framework for selecting AI approaches according to biosensor data characteristics and analytical requirements is summarised in Table 2.

### 3.3. Biosensor-Specific Use of Deep Learning Architectures

Biosensor inputs are typically represented as channel × time point sequences for physiological signals, intensity × wavelength/Raman shift vectors for spectroscopic data, and height × width × channel arrays for images. A typical CNN uses successive convolution–activation–pooling blocks, followed by classification or regression layers, whereas RNN/LSTM models process time step × feature sequences, and transformers use embedded sequences with multi-head self-attention and feed-forward layers [29,30,31,32,33]. Model depth and input length should be balanced against available training data, memory, latency, and deployment constraints [29,30,31,32,33].

**Table 2:** Practical selection of AI approaches for biosensor applications [14,28,31,32,33,34,35,36].

Biosensor Data Characteristic	Typical Analytical Task	Suitable AI Approaches	Advantages	Important Limitations
Low-dimensional electrochemical or tabular data	Calibration, classification, concentration prediction	Logistic regression, SVM/SVR, RF, XGBoost	Effective with relatively small datasets; lower computational burden	Performance depends on feature quality; may not capture complex spatial or temporal relationships
Continuous physiological time series data	Event detection, forecasting, longitudinal monitoring	1D-CNN, RNN, LSTM	Captures temporal or local signal patterns	RNN/LSTM processing is sequential; performance depends on windowing and sequence length
Images or spatial sensor maps	Image classification, feature localisation	CNN	Automated spatial feature extraction	Often requires larger labelled datasets and greater computation
Spectroscopic data, including Raman/SERS	Molecular classification, spectral pattern recognition	CNN, autoencoder, transformer-based models	Can analyse high-dimensional and overlapping spectral information	Risk of overfitting with small sample numbers; preprocessing strongly affects performance
Long or complex sequences	Long-range dependency modelling	Transformers	Captures relationships across distant signal regions	Self-attention can have high memory and computational requirements, limiting edge deployment
Unlabelled or weakly labelled sensor data	Representation learning, denoising, anomaly detection	Autoencoders, clustering, one-class methods	Reduces dependence on fully labelled datasets	Learned patterns may be difficult to interpret clinically
Multimodal biosensor data	Fusion of physiological, biochemical, imaging or contextual data	Multimodal neural networks, attention-based models	Integrates complementary information	Requires synchronised datasets and careful handling of missing or heterogeneous data

CNNs are well suited to biosensor data containing local spatial or temporal patterns. For a one-dimensional physiological signal, the input commonly consists of one or more sensor channels sampled across successive time points, with convolutional filters learning local waveform characteristics. For images or two-dimensional spectroscopic representations, convolution is applied across spatial or spectral dimensions. Pooling or strided convolution can reduce dimensionality before classification or regression. CNNs have consequently been applied to ECG-based arrhythmia detection and spectroscopic biosensing [29,30]. However, high internal classification accuracy should not by itself be considered evidence of clinical utility, particularly when external validation and heterogeneous clinical testing are absent.

RNNs and LSTMs are designed for sequential data in which current observations are influenced by preceding measurements. Biosensor inputs are generally represented as sequences of time points with one or more measured features at each time point. This makes these architectures applicable to continuous physiological signals, including ECG and glucose monitoring data [31,32]. LSTMs use gating mechanisms to retain or discard information over longer sequences, although their sequential computation may increase inference time compared with convolution-based approaches.

Transformers use self-attention to identify relationships between different positions within an input sequence and can therefore model long-range dependencies in high-dimensional biosensor data [33]. They may be useful for long physiological recordings, complex spectra, and multimodal sensing. Attention-based approaches have also been explored for SERS data [30,34]. However, conventional self-attention becomes increasingly computationally and memory intensive as sequence length increases. This is an important limitation for wearable and edge-based biosensors, where model compression, efficient architectures, or external processing may be required.

Autoencoders consist of an encoder that compresses input data into a latent representation and a decoder that reconstructs the original signal. In biosensing, this architecture can support signal denoising, anomaly detection, dimensionality reduction, and representation learning [35]. Variational and conditional autoencoders may additionally assist when available training data are limited, although synthetic or reconstructed data cannot substitute for independent clinical validation [36].

Thus, the relevance of a deep learning architecture should be assessed according to the biosensor signal, clinical task, available dataset, computational requirements, and validation strategy rather than model complexity alone.

### 3.4. Explainable AI and Clinical Decision Support

Interpretability remains an important consideration for AI-enabled biosensors, particularly when algorithmic outputs contribute to clinical decisions. Explainable AI (XAI) approaches, including Shapley Additive Explanations (SHAP), Gradient-weighted Class Activation Mapping (Grad-CAM), and Local Interpretable Model-Agnostic Explanations (LIME), can provide information about the features contributing to individual predictions [37].

In biosensing, XAI may help determine whether model predictions are driven by biologically or clinically plausible signal characteristics rather than artefacts or confounding variables. For example, SHAP-based approaches have been used to identify influential features in biosensor and wearable sensor classification models [38,39]. However, explainability should not be considered equivalent to model validity. An interpretable model may still perform poorly when applied to an external population, different sensor platform, or altered clinical environment.

Integration of AI-enabled biosensors into clinical decision support systems therefore requires more than high predictive accuracy. Appropriate external validation, calibration assessment, robustness across heterogeneous populations, clinically meaningful performance thresholds, transparent reporting, and continued performance monitoring are necessary before algorithmic outputs can be reliably incorporated into clinical workflows [37,40].

## 4. AI-Driven Biosensing Systems: From Signal to Decision

AI-enabled biosensing involves a sequential workflow in which raw transducer signals are digitised, preprocessed, analysed using computational models, and translated into clinically interpretable outputs. The reliability of this process depends not only on the predictive performance of the AI model but also on appropriate signal acquisition, data quality, preprocessing, computational efficiency, and the intended deployment environment. These factors become particularly important in wearable, point-of-care, and continuously monitoring biosensors, where latency, energy consumption, connectivity, and signal variability can directly influence system performance. The overall workflow from biosensor signal acquisition to AI-assisted interpretation and deployment is illustrated in Figure 2.

### 4.1. Signal Digitisation and Data Challenges

Raw biosensor signals must first be converted into digital representations suitable for computational analysis, typically using an analogue-to-digital converter (ADC). Sampling rate is an important design consideration because insufficient sampling may result in loss of relevant signal information, whereas unnecessarily high sampling rates increase data storage, computational, and energy requirements. In wearable ECG applications, sampling rates around 125 Hz have been reported to retain sufficient information for frequency domain feature extraction, although the optimal rate depends on the physiological signal and intended analytical task [41]. Similarly, Villa et al. demonstrated that an intermediate sampling rate of 20 Hz provided favourable performance for a CNN–LSTM-based wearable inertial system while reducing computational and storage requirements [42]. These findings highlight the importance of optimising sampling frequency for individual biosensing applications rather than applying uniformly high acquisition rates.

Following digitisation, the quality, quantity, and representativeness of the acquired data become critical determinants of AI performance. Biosensor datasets are frequently limited by small sample sizes, noisy or incomplete measurements, restricted representation of clinically relevant conditions, and variability in experimental settings. For example, an electrochemical biosensor dataset containing only 16 measurements distributed across five concentration levels illustrates data scarcity rather than class imbalance [43]. Such small datasets increase the risk of overfitting and may limit the generalisability of models to independent samples, different devices, or heterogeneous clinical populations.

Reliable labelling of biosensor data represents an additional challenge. Annotation of physiological events, disease states, or analyte concentrations may require expert interpretation or comparison with reference laboratory measurements, making the process resource-intensive and potentially susceptible to variability [44]. Furthermore, differences in sensor fabrication, biological matrices, experimental conditions, device characteristics, and measurement protocols can produce heterogeneous datasets that are difficult to compare across studies. Models trained under narrowly defined experimental conditions may consequently show reduced performance when transferred to different biosensor platforms or clinical environments [12,14].

Standardised data acquisition, adequate and representative datasets, clearly defined reference labels, and independent validation are therefore important prerequisites for developing generalisable AI-enabled biosensing systems.

### 4.2. Data Preprocessing and Feature Representation

Preprocessing is required to improve the quality and comparability of biosensor signals before they are used for AI model development. Depending on the sensing modality, preprocessing may include removal of artefacts, baseline correction, filtering, treatment of missing values and outliers, normalisation, and transformation of raw signals into representations suitable for subsequent analysis.

Conventional signal processing techniques remain useful for many biosensor applications, while ML- and DL-based approaches can provide additional capabilities when signals contain complex nonlinear noise or variable background interference [36]. Effective noise reduction and baseline correction are particularly important for continuously monitored signals, such as those generated by continuous glucose monitoring systems, where sensor drift and physiological variability may affect subsequent predictions [45].

Normalisation is also important when measurements are obtained from different devices, batches, patients, or experimental conditions. By reducing differences in signal scale or baseline characteristics, normalisation can improve comparability across measurements. Zhang et al. demonstrated a dynamic response approach that reduced false responses and manufacturing-related variability in biosensor measurements [43].

Feature extraction subsequently converts processed signals into informative representations for model development. Time domain, frequency domain, and time–frequency features can be selected according to the characteristics of the sensor output. For complex physiological or electrochemical signals, transformations such as short-time Fourier analysis may improve representation of temporal and spectral information [36].

Data augmentation may also be considered when datasets are limited. Generative approaches, including generative adversarial networks, can create synthetic examples that approximate characteristics of the available training data [44]. However, synthetically generated observations should be used cautiously because they do not replace independent experimental or clinical data and may reproduce biases present in the original dataset.

High-dimensional signals, including impedance spectra, voltammograms, Raman spectra, and multiplexed sensor outputs, may require dimensionality reduction before classification or regression. Principal component analysis (PCA) provides a linear approach for reducing correlated features, whereas autoencoders can learn nonlinear lower-dimensional representations [14,36]. In relatively small datasets, combining appropriate feature reduction with conventional ML may sometimes be more suitable than using highly complex deep learning architectures [46].

Thus, preprocessing should be selected according to the biosensor modality and analytical task rather than applied as a fixed pipeline. Importantly, all preprocessing procedures should be defined before final model evaluation and incorporated within the validation workflow to minimise information leakage and overly optimistic performance estimates.

### 4.3. Real-Time Inference and Resource-Constrained Deployment

One of the major potential advantages of AI-enabled biosensors is the ability to interpret incoming measurements rapidly enough to support clinically relevant decisions. Real-time performance, however, depends on more than classification accuracy. Latency, computational requirements, memory, power consumption, sampling frequency, and communication delays must also be considered when selecting and deploying an AI model.

Deep learning-assisted point-of-care platforms illustrate how computational interpretation can shorten the interval between signal acquisition and diagnostic output. For example, AI-assisted lateral flow analysis has demonstrated rapid interpretation compared with conventional manual assessment [47]. Nevertheless, reported processing times should be interpreted in relation to the complete diagnostic workflow, including sample preparation, signal acquisition, model inference, and reporting, rather than inference time alone.

Continuous monitoring creates additional challenges because wearable and implantable devices generate repeated or continuous streams of physiological and biochemical data. Cardiovascular monitoring, continuous glucose sensing, and other longitudinal applications require algorithms capable of detecting clinically relevant changes while limiting false alerts and computational burden [45,48]. Continuous monitoring systems may perform periodic or persistent inference, whereas event-triggered systems can activate processing after predefined signal changes or threshold events. The appropriate architecture depends on clinical purpose, urgency, energy availability, and acceptable latency.

Multiplexed wearable systems can simultaneously generate measurements from several physiological or biochemical targets [49]. AI may assist in integrating these signals and recognising temporal patterns that cannot be captured by isolated threshold measurements. However, analytical sensitivity or wide measurement ranges alone do not establish clinical benefit; the resulting output must be demonstrated to correspond to clinically meaningful concentrations, trends, or actionable events.

Tiny machine learning (TinyML) and other model compression strategies provide potential approaches for deploying AI on resource-constrained hardware. Techniques such as quantisation, pruning, and compact neural network architectures can reduce memory and computational requirements and facilitate on-device inference [50]. Edge-based ECG classification studies demonstrate the feasibility of implementing lightweight AI models on embedded platforms [51]. However, claims regarding ultra-low energy consumption should be interpreted cautiously because reported values may refer to individual inference operations or specific hardware components rather than the total power requirements of the complete biosensor system.

Real-time AI performance therefore involves a trade-off among accuracy, latency, energy consumption, memory, and robustness. Moreover, models that demonstrate high performance under controlled experimental conditions still require independent validation across devices, populations, and real-world operating environments before they can be considered clinically generalisable.

### 4.4. IoMT, Cloud, Edge Computing, and Federated Learning

Connected biosensors increasingly operate within the Internet of Medical Things (IoMT), where wearable, implantable, point-of-care, and home monitoring devices communicate with external computational infrastructure. IoMT enables biosensor data to be transmitted, aggregated, analysed, and integrated with remote monitoring or clinical information systems [52].

Cloud computing provides substantial storage and computational capacity and is particularly useful for large-scale model training, longitudinal data analysis, population-level monitoring, and computationally demanding algorithms that cannot be efficiently executed on a local biosensor [53,54]. However, cloud-based processing can introduce communication latency, bandwidth requirements, dependence on network availability, and privacy and cybersecurity concerns.

Edge computing represents a complementary rather than superior alternative to cloud computing. It places computation closer to the point of data generation, including on microcontrollers, field-programmable gate arrays, smartphones, gateways, or other local processing devices [55]. By reducing the need to continuously transmit raw biosensor data to remote servers, edge processing can decrease latency, bandwidth requirements, and exposure of sensitive information. It may therefore be particularly suitable for time-sensitive point-of-care and wearable applications.

The optimal architecture may combine device, edge, fog, and cloud resources rather than relying exclusively on one computational layer. Lightweight preprocessing and inference can occur locally, while computationally intensive model training, long-term storage, population-level analytics, or periodic model updating can be performed using cloud infrastructure. Such distributed architectures allow for computational resources to be selected according to latency, memory, energy, connectivity, and privacy requirements.

Federated learning provides an additional strategy for distributed AI development by enabling models to be trained across multiple devices or institutions without routinely transferring the underlying raw patient data to a central repository [56]. This approach may be valuable for multi-institutional biosensor datasets where direct data sharing is constrained by privacy, governance, or regulatory requirements. However, federated learning does not itself guarantee privacy or model generalisability. Differences in patient populations, sensor platforms, local datasets, network connectivity, and data distributions may affect model convergence and performance, and additional privacy and security safeguards remain necessary.

Together, IoMT, edge computing, cloud computing, TinyML, and federated learning provide complementary components of the deployment infrastructure for AI-enabled biosensors. Their selection should be guided by the intended clinical application rather than technological novelty, with consideration of latency, computational resources, energy requirements, privacy, interoperability, and the need for continued model validation.

## 5. Performance Evaluation, Clinical Validation and Reporting Standards

Evaluation of an AI-enabled biosensor requires assessment of the complete sensor–algorithm system rather than either component in isolation. Conventional analytical characteristics—including sensitivity, selectivity, linearity, dynamic range, precision, reproducibility, interference and limit of detection (LoD)—remain fundamental because they describe the performance of the physical sensing platform. AI introduces additional requirements related to discrimination, calibration, generalisability, uncertainty, computational performance and the clinical consequences of model outputs. Accordingly, high predictive accuracy alone should not be regarded as sufficient evidence of a clinically reliable AI-enabled biosensor. Contemporary guidance for medical AI similarly emphasises transparent specification of intended use, target populations, input and output data, model evaluation and implementation context [57,58,59,60].

For translational assessment, performance can usefully be considered at three interconnected levels. Analytical validity concerns whether the integrated sensing system reliably measures the intended signal or analyte under specified conditions. Clinical validity concerns whether the resulting measurement or prediction accurately identifies or predicts the clinical state for which the system is intended. Clinical utility goes further by asking whether use of the system changes clinical decisions, workflow, accessibility, treatment or patient outcomes in a beneficial manner. This distinction is important because much of the AI biosensor literature remains at the analytical or retrospective model development stage, whereas live clinical evaluation requires evidence about human–AI interaction, workflow and consequences of use. DECIDE-AI was developed to improve reporting of early-stage AI evaluations in live clinical settings, while CONSORT-AI addresses later interventional trials [59,60].

### 5.1. Analytical and Algorithmic Performance

Performance metrics should be selected according to the intended analytical and clinical task. For classification systems, relevant measures may include sensitivity, specificity, precision, recall, F1-score, receiver operating characteristic area under the curve (AUROC), and positive and negative predictive values. Accuracy should not be used as the sole metric, particularly when classes are imbalanced. Predictive values should also be interpreted in the context of disease prevalence and the intended use population. TRIPOD+AI requires transparent reporting of model development and evaluation procedures and was explicitly designed for prediction models developed with either regression or machine learning methods [57].

Headline performance values require contextualisation. A model reporting 98–100% accuracy cannot be interpreted meaningfully without information on the number of biologically independent participants or specimens, class distribution, data partitioning, reference standard, confidence intervals and external evaluation. Repeated spectra, signal windows, images or heartbeats from the same patient are not equivalent to independent patients. DOME specifically warns that overlap between model development and evaluation data can create an illusion of generalisation and recommends clearly separating training and test data in supervised biological ML studies [61].

For regression applications—such as estimation of glucose concentration, metabolite concentration or another continuous physiological quantity—correlation alone is insufficient. Error magnitude, agreement, calibration and performance across the clinically relevant measurement range should also be evaluated. A model can discriminate between low and high values while remaining systematically biased in the absolute quantity that ultimately determines a clinical decision. TRIPOD+AI likewise treats calibration and model performance evaluation as core elements of transparent prediction model reporting [57].

### 5.2. Limit of Detection and Clinical Actionability

The LoD of a biosensor is fundamentally related to the physicochemical properties of the recognition element and transducer, background noise, sample matrix and measurement conditions. AI should therefore not be described as a “digital amplifier” that intrinsically lowers the physical LoD of a transducer [4,14,16].

Machine learning and deep learning algorithms can nevertheless improve the interpretation of weak or complex sensor outputs by modelling background variation, denoising measurements, extracting multivariate features or recognising patterns close to the noise floor. In that sense, AI may improve the effective or operational detectability of the complete sensor–algorithm system, but a lower LoD should only be claimed when the integrated system’s LoD has been experimentally re-estimated and validated. Improved classification accuracy by itself does not demonstrate a lower analytical LoD. The recent literature on ML-assisted electrochemical sensing supports using ML to handle interference, fouling, low signal-to-noise conditions, and complex multivariate sensor outputs, but these computational gains should be distinguished from the underlying transducer physics [14,46].

More importantly, judge the relevance of LoD against the intended clinical decision. Table 3 presents representative examples illustrating the relationship between analytical LoD and clinically relevant decision points.

Several established frameworks now provide study design-specific guidance for medical AI. TRIPOD+AI provides reporting recommendations for studies developing or evaluating clinical prediction models using regression or machine learning methods [57]. STARD-AI addresses diagnostic accuracy studies involving AI [58], while DECIDE-AI addresses early-stage clinical evaluation of AI-based decision support systems in live healthcare settings [59]. CONSORT-AI and SPIRIT-AI extend clinical trial reporting and protocol guidance, respectively, to interventions involving AI [60,62]. MI-CLAIM provides minimum information recommendations for transparent clinical AI modelling [63], and DOME addresses data, optimisation, model development and evaluation in supervised machine learning studies in biology [61]. QUADAS-AI has also been proposed as an AI-specific extension for risk-of-bias and applicability assessment in diagnostic accuracy research, although the currently published source is its development protocol and it should not be described as a finalised tool [64].

**Table 3:** Examples illustrating the distinction between analytical LoD and clinically relevant decision thresholds.

Analyte	Example of a Clinically Relevant Concentration or Decision Point	Correct Interpretation for Biosensor Development
Glucose	Diabetes diagnostic criteria include fasting plasma glucose ≥126 mg/dL (7.0 mmol/L) and 2-h plasma glucose ≥200 mg/dL (11.1 mmol/L) during a 75-g OGTT [65].	Once a glucose sensor performs reliably well below the required diagnostic or monitoring range, further orders-of-magnitude reductions in LoD may be less important than calibration accuracy, dynamic range, lag, reproducibility and resistance to matrix effects.
Cardiac troponin	Myocardial injury is defined by cardiac troponin exceeding the assay-specific 99th-percentile upper reference limit; myocardial infarction additionally requires clinical evidence of acute ischemia [66].	There is no single universal troponin concentration that all biosensors should target. Analytical performance must be connected to an assay-specific validated clinical decision limit.
Serum lactate	In Sepsis-3, lactate >2 mmol/L is part of the septic-shock clinical construct when accompanied by vasopressor-dependent hypotension despite adequate volume resuscitation [67].	Very low lactate LoDs are not themselves evidence of superior sepsis diagnosis. Reliable measurement around clinically relevant concentrations, rapid turnaround and appropriate clinical context are more important.

These frameworks are complementary rather than interchangeable. STARD-AI advises investigators to choose the guideline that matches the primary study aim and notes that TRIPOD+AI is more appropriate when the study develops or evaluates a multivariable prediction model [57,58].

The remaining challenge is therefore not simply the absence of harmonised guidance, but inconsistent adoption of existing reporting and evaluation frameworks and the need to integrate AI-specific standards with analytical biosensor validation. Studies should report dataset provenance, numbers of biologically independent participants or specimens, class distributions, preprocessing, participant- or specimen-level partitioning, reference standards, model architecture and version, hyperparameter selection, calibration, uncertainty, external evaluation, subgroup performance and clinically relevant operating thresholds. Selection of a reporting framework should follow study design—for example, TRIPOD+AI for prediction models, STARD-AI for diagnostic accuracy studies, DECIDE-AI for early live clinical evaluation and CONSORT-AI/SPIRIT-AI for clinical trials [57,58,59,60,61,62,63,64].

## 6. Clinical Applications and Translational Evidence

AI-enabled biosensors are being investigated for infectious diseases, cancer, metabolic disorders, cardiovascular disease and longitudinal physiological monitoring. Their translational maturity, however, varies substantially. Evidence ranges from algorithm development using benchmark datasets or repeated measurements from a small number of specimens, through independent external testing, to prospective studies in real clinical workflows. Clinical applications should therefore be discussed not only by disease area but also by evidence maturity.

### 6.1. Infectious Diseases

AI-assisted optical and spectroscopic biosensing is particularly relevant to infectious disease diagnostics because Raman and SERS measurements generate high-dimensional molecular fingerprints whose interpretation may benefit from computational pattern recognition. Nevertheless, the number of recorded spectra should not be confused with the number of biologically independent isolates or patients [30,34,45].

Lyu et al. analysed 121 clinical Klebsiella pneumoniae isolates—21 polymyxin-resistant, 50 carbapenem-resistant and 50 carbapenem-sensitive—with 64 SERS spectra generated per strain. A CNN with an attention mechanism achieved 99.46% reported accuracy and 98.87% in five-fold cross-validation [34].

The result shows strong within-study discrimination but should be presented with its sample structure: thousands of spectra came from only 121 independent isolates. Repeated spectra improve isolate characterisation but do not create thousands of independent clinical subjects.

A more advanced example is the 2026 SERS–deep learning study of Hassan et al., which assembled a primary dataset from 653 analyte-enriched blood samples and tested an independent blind cohort of 70 samples. The model achieved 99.67% accuracy for binary sepsis recognition and 98.84% for six-class pathogen identification during sample-contained cross-validation; external pathogen-typing performance was 98.28% [68].

This design provides stronger evidence of generalisability than internal cross-validation alone. However, the specimens underwent analyte enrichment, and the study does not establish that deployment improves antimicrobial selection, time to effective treatment, mortality or health system costs. Those questions require prospective clinical evaluation.

Thus, infectious disease evidence should be described as progressing from spectral discrimination → independent specimen validation → prospective clinical utility, rather than assuming that high internal classification accuracy already constitutes a validated point-of-care diagnostic.

### 6.2. Cancer and Molecular Diagnostics

Cancer provides a strong rationale for AI-assisted biosensing because clinically useful information may reside in combinations of proteins, nucleic acids, extracellular vesicle signals or complex spectral fingerprints rather than in a single biomarker. Recent work illustrates both the opportunities and the current translational gap.

A 2025 electrochemical study of oral squamous cell carcinoma used salivary biomarkers with supervised ML; among the tested approaches, a combined biomarker AdaBoost analysis reached approximately 76% accuracy for identifying metastatic samples. The authors themselves identify the need for a larger cohort before clinical validation [69].

This is an important example for the review because it demonstrates that clinically relevant studies need not report near-perfect accuracy to be scientifically informative.

Other 2025 studies have moved toward multiplexed clinical biosensing. A bimodal extracellular vesicle circRNA analyser combined fluorescence and electrochemical outputs with ML to distinguish early gastric cancer from healthy donors in a clinical cohort [70].

An AI-guided SERS platform using serum exosome molecular fingerprints reported high discrimination across several cancer types, including early-stage disease, but this promising retrospective performance still requires prospective, independently replicated validation before population screening claims are justified [71].

The critical translational question, therefore, is not whether AI can fit a complex biosensor dataset. It is whether the selected biomarkers and model retain performance across independent populations, disease stages, benign mimics, inflammatory conditions, laboratories and device batches and whether the result changes an established clinical pathway.

The LoD argument is especially relevant in oncology. An extremely low LoD is useful only when the additional detectable concentration range contains clinically informative disease signal. Once the system can quantify the relevant biomarker range, improvements in multianalyte discrimination, specificity, between-batch reproducibility, calibration and external validity may contribute more to clinical usefulness than further orders-of-magnitude reductions in LoD [14,45,57,58].

### 6.3. Metabolic and Cardiovascular Monitoring

Metabolic and cardiovascular applications illustrate a different advantage of AI-enabled sensing: the ability to analyse longitudinal physiological signals rather than isolated biochemical measurements.

Chiu et al. used personalised one-class SVM models to detect dysglycaemia from ambulatory lead-II ECG data in the MIMIC-III database. The reported AUROC was 0.92 ± 0.09 for a single heartbeat and increased to 0.97 ± 0.06 after 10 s majority voting [72]. 

These findings support the feasibility of extracting glucose-associated physiological patterns from ECG, but the personalised and retrospective design should not be described as equivalent to a prospectively validated non-invasive glucose monitor.

A small wearable gait study similarly illustrates why sample size must accompany headline accuracy. Kim et al. analysed inertial sensor gait data from 42 women older than 65 years; XGBoost achieved 88.69% accuracy for osteopenia, and random forest achieved 93.75% for sarcopenia [39].

The appropriate interpretation is proof of concept rather than established population-level screening.

At the other end of the maturity spectrum, Adedinsewo et al. conducted a pragmatic randomised clinical trial of AI-guided cardiomyopathy screening in an obstetric population across six hospitals in Nigeria. Among 1195 participants completing the baseline visit, AI-enabled digital stethoscope screening detected left ventricular systolic dysfunction in 24/587 participants in the intervention arm compared with 12/608 in usual care, corresponding to an odds ratio of 2.12 (95% CI 1.05–4.27) [73].

Unlike retrospective accuracy studies, this trial examined an AI-enabled sensing intervention in an actual clinical pathway and was prospectively registered [73]. It therefore represents a substantially higher level of translational evidence.

These examples demonstrate why evidence maturity should be the organising axis: benchmark accuracy, internal cross-validation, external validation and prospective clinical effectiveness answer different questions and should not be presented as equivalent levels of evidence.

### 6.4. From Analytical Performance to Clinical Utility

AI-enabled biosensors should be presented as potentially complementary to, rather than inherently superior replacements for, established laboratory diagnostics. PCR, culture, immunoassays and central laboratory chemistry offer mature reference procedures for many clinical questions, whereas biosensors may provide advantages when decentralised, rapid, repeated or continuous measurement is specifically valuable.

These properties depend on the assay, sample preparation, calibration, connectivity, operator requirements and clinical setting. A rapid inference step does not necessarily mean that the complete test, from sample collection through preparation to result interpretation, is rapid.

Compared with centralised laboratory diagnostics, AI-enabled biosensors may provide advantages in applications requiring decentralised, repeated or continuous measurement and automated interpretation. However, their value depends on the intended use and should not be inferred solely from miniaturisation, model accuracy or analytical sensitivity. Comparison with conventional diagnostics should consider sample preparation, reference standard agreement, turnaround time for the complete workflow, calibration, reproducibility, infrastructure requirements, failure modes, cost and the clinical consequences of false positive and false negative results. Accordingly, AI-enabled biosensors should be evaluated as complementary diagnostic or monitoring tools whose usefulness must be demonstrated within a defined clinical pathway [11,12].

### 6.5. Critical Evidence Synthesis

The studies summarised in Table 4 were selected as illustrative examples rather than as an exhaustive systematic sample. Selection was restricted to studies in which an identifiable sensing platform generated the analysed signal and an explicit ML/DL method was applied to the sensor-derived data. Examples were chosen to span distinct sensing modalities and, importantly, different levels of translational maturity, from benchmark or proof-of-concept evaluation through independent external testing to prospective clinical evaluation.

### 6.6. Publication Bias and Evidence Limitations

The literature on AI-enabled biosensors is particularly susceptible to selective emphasis on highly performing models. Studies reporting novel architectures or very high accuracy may attract greater attention than negative, modest or inconclusive findings, and model development studies can test multiple preprocessing procedures, feature sets and algorithms before highlighting the best result. Therefore, the published distribution of performance should not automatically be interpreted as the distribution expected during independent real-world deployment.

Reporting frameworks can reduce, but not eliminate, this problem. CONSORT-AI requires detailed reporting of AI interventions and error cases in clinical trials, while SPIRIT-AI establishes AI-specific protocol reporting requirements before trial results are known [60,62].

A 2024 assessment of 65 AI randomised trials found that only 10 explicitly reported using CONSORT-AI and identified persistent under-reporting of algorithm versions, accessibility, and study protocols, showing that the challenge is partly uptake of standards, not simply their absence [74].

Prospective registration, protocol publication and prespecification of primary outcomes and analytical plans should be encouraged for clinical evaluations of AI-enabled biosensors. Reports should disclose unsuccessful or alternative model comparisons where relevant and should distinguish prespecified analyses from exploratory optimisation. For evidence synthesis, registered studies and protocols may also help identify completed or ongoing evaluations that have not produced highly positive journal publications. These measures would reduce selective reporting and provide a more realistic assessment of translational readiness [60,62,74].

## 7. Challenges, Regulation, Ethics and Future Directions

The principal challenge in AI-enabled biosensing is no longer simply achieving high analytical sensitivity or model accuracy. Translation requires the sensing chemistry, hardware, data pipeline, AI model, deployment environment and clinical workflow to remain reliable as an integrated system.

### 7.1. Sensor and Data Limitations

Biofouling, matrix interference, sensor drift, device-to-device variation and environmental changes remain fundamental limitations because AI operates on the signals generated by the physical sensor. If biological adsorption progressively attenuates an electrode response, or motion corrupts a wearable signal, the distribution of the model input can differ substantially from that encountered during development [13,18,19,20].

AI-based calibration or denoising may compensate for predictable forms of variation, but this capability should not be overstated. Algorithms cannot restore information that was never captured because of severe fouling, saturation, unstable recognition chemistry or device failure. Sensor engineering, calibration, and computational correction therefore need joint validation [13,14,36].

Data scarcity creates a separate problem. Biosensor studies frequently produce many signal segments or replicate spectra from comparatively few independent participants or specimens. The apparent size of a machine learning dataset can therefore far exceed its biological sample size. This distinction should be explicitly reported. DOME identifies data separation and model evaluation as central components of reproducible biological ML [61].

Domain shift is another important translational problem: differences in populations, prevalence, laboratories, device batches, acquisition protocols or clinical workflows can alter model performance outside the development setting. This is why external evaluation should be prioritised over increasingly elaborate internal cross-validation once a model has been developed [57,58,59,61].

### 7.2. Interpretability, Uncertainty and Human Oversight

Explainable AI may help identify which signal components contribute to a prediction, but explanation should not be equated with validation. A SHAP plot can make a prediction more inspectable while the underlying model remains biased, poorly calibrated or non-generalisable. Explainability should therefore complement—not replace—external validation, calibration, error analysis and clinical evaluation [37].

For clinical decision support, the role of the human user should also be explicit. CONSORT-AI requires reporting how AI outputs contribute to clinical decision-making and emphasises human–AI interaction [60].

DECIDE-AI similarly focuses on live clinical evaluation and how AI systems interact with users and workflows [59].

This is also consistent with current regulation. For high-risk AI systems, the EU AI Act requires effective human oversight designed to prevent or minimise risks to health, safety and fundamental rights [75].

Thus, clinically deployed AI biosensors should communicate not only a prediction but, where appropriate, confidence, limitations, relevant quality control information and circumstances in which users should disregard or confirm the output.

### 7.3. Regulation and Lifecycle Governance

An AI system is high-risk through the product route when both conditions are met: the AI system is itself a product or safety component covered by the Union harmonisation legislation, and the relevant product is required to undergo third-party conformity assessment. Medical devices and in vitro diagnostic medical devices are among the product categories covered by this framework.

The regulatory classification of an AI-enabled biosensor depends on its intended purpose, its role in the medical device, and the applicable conformity assessment pathway; CE marking alone should not be used as a synonym for high-risk AI classification under the EU AI Act [75].

Regulation also increasingly addresses changes occurring after initial deployment. The Good Machine Learning Practice principles, initially developed by the FDA, Health Canada, and the UK MHRA and subsequently formalised by the IMDRF, adopt a total product lifecycle perspective for AI/ML-enabled medical devices [76].

The FDA has final guidance addressing Predetermined Change Control Plans for AI-enabled device software, allowing for planned modifications to be described together with methods for development, validation and impact assessment [77].

This is particularly relevant to AI-enabled biosensors because modifications to sensor hardware, preprocessing, firmware or model parameters may alter the behaviour of the integrated system.

Thus, future AI biosensor development should treat model versioning, sensor hardware changes, post-deployment monitoring and retraining as part of a controlled lifecycle rather than assuming that a model validated once remains valid indefinitely.

### 7.4. Privacy, Cybersecurity, Bias and Equity

Connected biosensors may continuously transmit physiological or biochemical information, making cybersecurity a patient safety issue as well as a data privacy issue. The FDA’s current cybersecurity guidance recommends cybersecurity considerations in device design, labelling and premarket documentation, and its February 2026 final guidance addresses devices with cybersecurity risk [78].

The practical relevance is not theoretical: FDA communications in 2025 documented vulnerabilities in connected patient monitors that could permit unauthorised control and patient data exfiltration [79].

Federated learning can reduce the need to pool raw data centrally, but it should not be described as guaranteeing privacy. Privacy-preserving architecture does not remove requirements for authentication, cybersecurity, governance, access control or secure model update procedures [56,78].

Algorithmic equity should also be evaluated empirically. Aggregate accuracy may conceal poor performance in clinically important subgroups. Data used for development should therefore reflect the intended population, and subgroup analyses should be planned where age, sex, ethnicity, comorbidities, disease severity, skin characteristics, sensor placement or other factors could plausibly influence the sensing signal or AI output. FDA transparency principles emphasise communicating intended populations, performance, risks and bias management information throughout the lifecycle [80].

### 7.5. Future Directions

The next phase of AI-enabled biosensing should prioritise robustness, adaptation and clinical usefulness rather than algorithmic complexity alone.

Adaptive calibration and domain adaptation approaches may help systems cope with predictable changes in sensor response, but adaptive behaviour must be controlled so that model updates do not introduce unvalidated changes. Current FDA lifecycle and PCCP approaches illustrate the broader regulatory shift toward predefined plans for how AI-enabled medical software may change after deployment [77].

Edge and cloud computing should also be described as complementary computational tiers. Edge processing can support lower-latency local inference and reduce continuous data transmission, whereas cloud infrastructure can support computationally intensive training, longitudinal analytics and large-scale model management [53,55].

Multimodal biosensing is another important direction. Combining biochemical signals with ECG, photoplethysmography, motion, imaging, or clinical variables may provide more informative representations than a single marker. Recent gastric cancer work, for example, has combined dual-mode circRNA sensing with ML-based multiparametric analysis, illustrating the movement from isolated single-analyte measurements toward integrated molecular signatures [70].

However, increasing dimensionality is not automatically beneficial. Multimodal systems create additional risks of missing data, overfitting, calibration mismatch and reduced portability between devices. More complex architectures should therefore be justified by demonstrable improvement over simpler baselines.

The most important future direction is stronger evidence generation. A pragmatic multicentre AI-guided cardiomyopathy trial has already demonstrated that sensor-plus-AI systems can be evaluated in real clinical workflows rather than only retrospectively [73].

Future biosensor studies should increasingly progress along the same evidence pathway: analytical validation → independent external evaluation → prospective workflow evaluation → comparison with standard care → patient- or system-level clinical utility.

## 8. Conclusions

Artificial intelligence can extend biosensor capabilities through signal processing, feature extraction, multivariate interpretation, calibration, pattern recognition and decision support. However, increasingly complex models, near-perfect internal accuracy and progressively lower analytical LoDs should not be treated as endpoints in themselves. The clinical value of an AI-enabled biosensor depends on whether the integrated sensor–algorithm system performs reproducibly in relevant samples and populations and whether its output corresponds to a concentration, physiological state or risk threshold that can meaningfully influence clinical decisions.

Future progress therefore requires a shift from proof-of-concept performance toward externally evaluated, appropriately calibrated, robust and clinically useful systems. Existing reporting frameworks—including TRIPOD+AI, STARD-AI, DECIDE-AI, CONSORT-AI and SPIRIT-AI—already provide substantial guidance for transparent evaluation at different stages of the clinical AI evidence pathway, while MI-CLAIM and DOME provide additional modelling and reproducibility guidance.

Integration of these standards with rigorous biosensor analytical validation, prospective multicentre studies, lifecycle governance, cybersecurity and clinically meaningful endpoints will be essential for translating AI-enabled biosensing from impressive laboratory demonstrations into dependable diagnostic and monitoring systems.

## Figures and Tables

**Figure 1:**
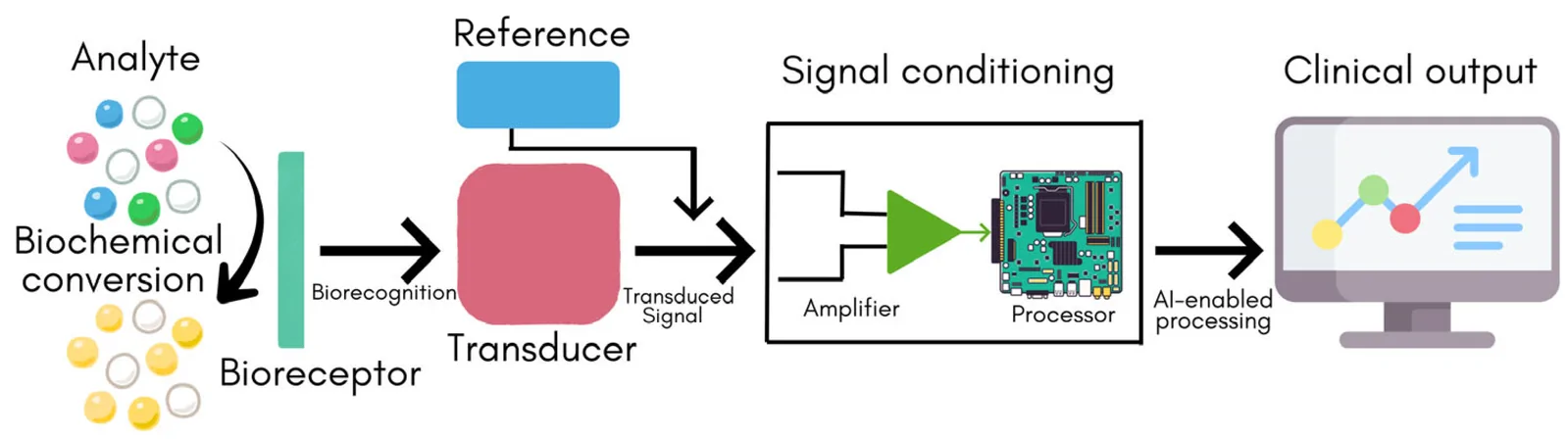
General architecture of a biosensor and potential integration of artificial intelligence within the biosensing pipeline. A target analyte interacts with a biological recognition element, and the resulting biorecognition event is converted into a measurable signal by the transducer. Signal conditioning, analogue-to-digital conversion, and computational processing subsequently generate an interpretable output. AI-based methods may support preprocessing, calibration, feature extraction, regression or classification, anomaly detection, and interpretation of biosensor-derived data.

**Figure 2:**
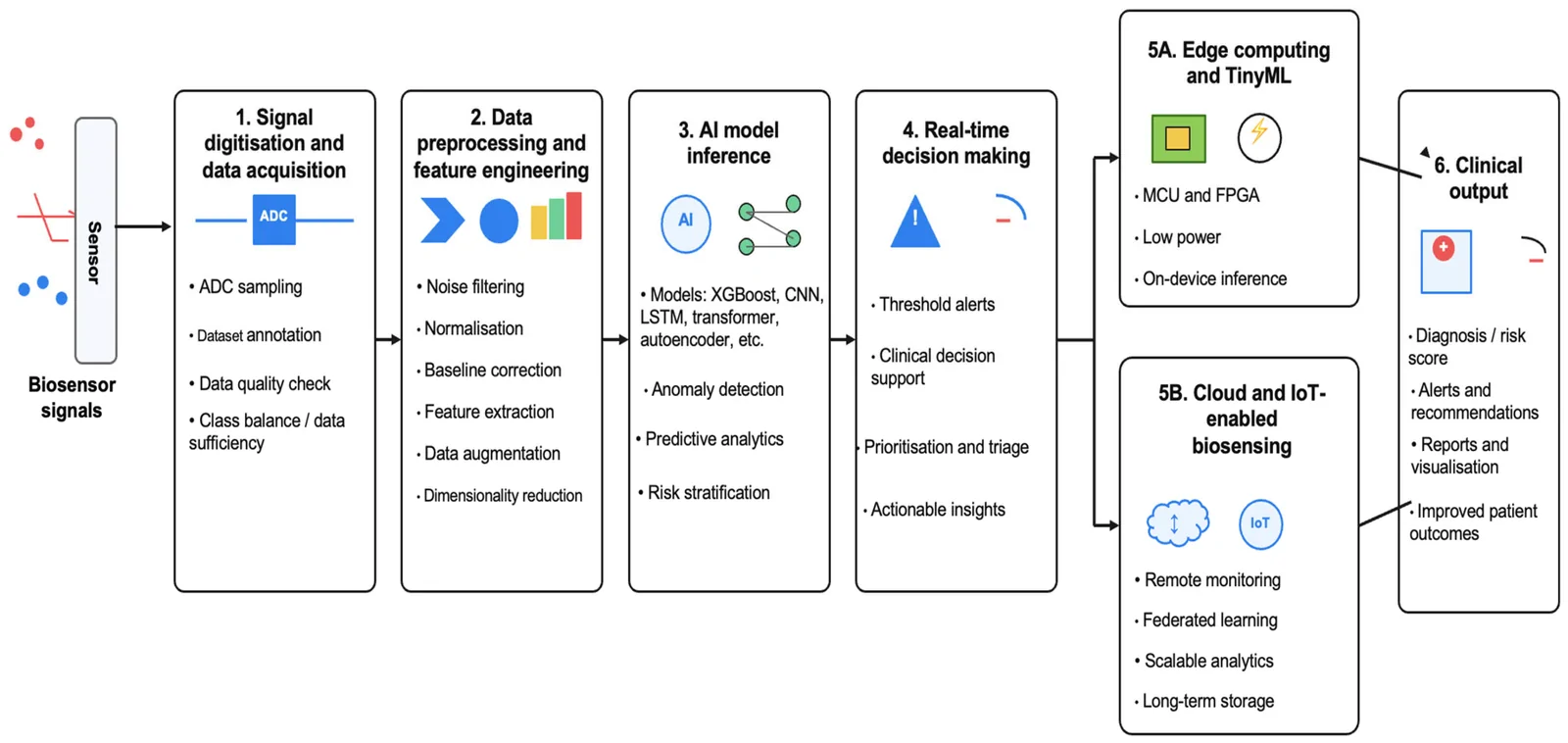
**Workflow of an AI-enabled biosensing system.** Raw biosensor signals are digitised and preprocessed before analysis using machine learning or deep learning models. Depending on the intended application and computational requirements, inference may occur locally through edge computing or TinyML, or through connected cloud and IoMT infrastructure. Federated learning may support distributed model development without centralising raw patient data. The final output should provide clinically interpretable information or alerts, while successful deployment depends on robust data acquisition, appropriate validation, interoperability, privacy, and computational efficiency.

**Table 1:** Biosensor transduction modalities and their relevance to artificial intelligence [1,4,11,12,14,16,17].

Transduction Modality	Typical Sensor Output	Major Data-Related Challenges	Potential Role of AI
Electrochemical	Current, potential, voltage, impedance or voltametric response	Electrical noise, baseline drift, nonlinear response, electrode fouling, matrix interference and device-to-device variability	Denoising, drift correction, calibration, feature extraction, regression and classification
Optical	Fluorescence, absorbance, reflectance, refractive index changes or images	Background interference, illumination variability, overlapping features and high-dimensional image data	Background correction, image analysis, feature extraction, regression and classification
Spectroscopic *	Raman, SERS or related spectra	High dimensionality, weak signals, baseline variation and overlapping spectral signatures	Baseline correction, dimensionality reduction, feature extraction, pattern recognition and classification
Piezoelectric/mechanical	Frequency, resonance, mass-related or mechanical response changes	Environmental interference, drift and temporal variability	Filtering, regression, temporal modelling and anomaly detection
Thermal/calorimetric	Temperature or heat-flow changes	Ambient temperature dependence, baseline drift and low-amplitude responses	Baseline correction, regression, classification and anomaly detection
Multimodal	Combined biochemical, physiological, image or spectral signals	Data heterogeneity, different sampling frequencies, temporal synchronisation, missing data and unequal signal quality	Data fusion, multimodal representation learning, classification and predictive modelling

* Spectroscopic biosensors may be considered a subset of optical biosensors but are shown separately because their high-dimensional spectral output creates distinct computational requirements.

**Table 4:** Representative AI-enabled sensing studies organised by evidence maturity.

Application and Sensing Modality	Independent Biological/Clinical Data	AI Approach	Evaluation Design	Key Reported Result	Translational Interpretation
Portable ECG arrhythmia sensing, Panwar et al. 2025 [29]	Model evaluation based on the MIT-BIH benchmark dataset	1D CNN	Benchmark/internal evaluation	98.35% binary; 99.3% multiclass accuracy	Engineering proof of concept. High benchmark performance does not establish prospective device performance in an independent clinical population.
Wearable gait sensing, Kim et al. 2022 [39]	42 older women	XGBoost/RF + SHAP	Small clinical cohort/cross-validation	88.69% osteopenia; 93.75% sarcopenia accuracy	Small clinical proof of concept. Sample size limits precision and generalisability.
ECG-based dysglycaemia detection, Chiu et al. 2023 [72]	Retrospective ambulatory ECG and glucose measurements	Personalised one-class SVM	Retrospective/personalised evaluation	AUROC 0.92 per heartbeat; 0.97 after 10-s voting	Retrospective feasibility. Needs independent prospective wearable validation.
SERS antimicrobial resistance phenotyping, Lyu et al. 2023 [34]	121 *K. pneumoniae* isolates; 64 spectra/isolate	CNN + attention	Five-fold cross-validation	99.46% accuracy; 98.87% CV robustness	Clinical-isolate proof of concept. Repeated spectra are not independent isolates; no separate external isolate cohort reported.
SERS sepsis/pathogen identification, Hassan et al. 2026 [68]	653 primary + 70 external blind samples	SuperRamanNet	Sample-contained CV + independent blind cohort	98.28% external pathogen-typing accuracy	External validation. Stronger generalisability evidence, but analyte enrichment and lack of prospective outcome testing remain limitations.
AI-guided obstetric cardiomyopathy screening, Adedinsewo et al. 2024 [73]	1195 baseline participants across six hospitals	AI-enabled digital stethoscope/AI-ECG	Pragmatic randomised clinical trial	LV systolic dysfunction detected in 4.1% vs. 2.0%; OR 2.12, 95% CI 1.05–4.27	Prospective multicentre randomised implementation trial; strong translational evidence, although generalisability beyond the studied obstetric population requires further evaluation.

## Data Availability

No new datasets were generated or analysed in this review.

## Supplementary Materials

The following supporting information can be downloaded at: https://www.mdpi.com/article/10.3390/bios16100537/s1, Table S1: Literature search strategy and study selection framework.
