# Supplementary material for: Advancing Biosensing Through Artificial Intelligence: Overcoming Conventional Limitations Towards Intelligent Diagnostic Systems
Source: Biosensors (Basel). 2026 Sep 25;16(10):537. doi: 10.3390/bios16100537 (PMC13702692; doi:10.3390/bios16100537)
Supplement: Supplementary file 1 [file biosensors-16-00537-s001.zip › biosensors-4492314-supplementary.pdf]

**Supplementary Table S1. Literature-search strategy and study-selection framework**

| Item | Description |
|---|---|
| Review type | Structured narrative review with targeted critical appraisal of representative original studies |
| Databases | PubMed/MEDLINE, Scopus and Web of Science |
| Supplementary searching | Google Scholar; backward reference-list screening; forward citation tracking of key primary studies and major reviews |
| Search period | Database inception to 31 August 2026 |
| Recent-literature priority | Literature from 2022–2026 was prioritized for AI architectures, AI-enabled biosensing, validation, TinyML/edge deployment, federated learning and medical-AI reporting standards |
| Historical literature | Older landmark references were retained where historically or methodologically foundational |
| Core biosensor concepts | “biosensor”, “biosensing”, “wearable sensor”, “electrochemical biosensor”, “optical biosensor”, “SERS”, “Raman”, “point-of-care” |
| AI concepts | “artificial intelligence”, “machine learning”, “deep learning”, “CNN”, “RNN”, “LSTM”, “transformer”, “autoencoder”, “TinyML”, “edge computing”, “federated learning” |
| Translational concepts | “clinical validation”, “external validation”, “calibration”, “generalizability”, “diagnostic performance”, “limit of detection”, “clinical utility”, “reference standard” |
| Inclusion for critical appraisal | Peer-reviewed original studies integrating a biosensor or sensor-derived physiological/biochemical signal with an identifiable AI/ML method and providing sufficient information on sample/dataset size, validation strategy and performance |
| Exclusion from critical appraisal | Non-AI biosensor studies; purely computational studies without sensor-derived input; promotional or commercial material; studies with insufficient methodological or validation information |
| Study-selection rationale | Representative studies were selected to cover different sensing modalities, clinical applications and evidence maturity: proof-of-concept/internal validation → clinical sample/small cohort → external validation → prospective clinical evaluation |
| Additional authoritative sources | Clinical guidelines, regulatory documents and reporting standards were obtained from authoritative bodies and original guideline publications, including WHO, IUPAC, FDA, EU AI Act, TRIPOD+AI, STARD-AI, DECIDE-AI, CONSORT-AI, SPIRIT-AI, MI-CLAIM and DOME |

| | |
|---|---|
| Evidence synthesis | Qualitative narrative synthesis; no quantitative meta-analysis because of substantial heterogeneity in sensor platforms, analytes, datasets, AI models, validation designs and outcomes |
